# Ischemic Postconditioning Attenuates Myocardial Ischemia-Reperfusion-Induced Acute Lung Injury by Regulating Endoplasmic Reticulum Stress-Mediated Apoptosis

**DOI:** 10.21470/1678-9741-2021-0043

**Published:** 2023

**Authors:** Aimei Li, Siyu Chen, Jianjiang Wu, Jiaxin Li, Jiang Wang

**Affiliations:** 1 Department of Anesthesiology, the First Affiliated Hospital of Xinjiang Medical University, Xinjiang, China.

**Keywords:** Alveolar Epithelial Cells, Ischemia-Reperfusion, Acute Lung Injury, Coronary Vessels, Apoptosis, Carrier Proteins, Ischemic Postconditioning

## Abstract

**Objective:**

To explore the effect of ischemic postconditioning on myocardial ischemia-reperfusion-induced acute lung injury (ALI).

**Methods:**

Forty adult male C57BL/6 mice were randomly divided into sham operation group (SO group), myocardial ischemia-reperfusion group (IR group), ischemic preconditioning group (IPRE group) and ischemic postconditioning group (IPOST group) (10 mice in each group). Anterior descending coronary artery was blocked for 60 min and then reperfused for 15 min to induce myocardial IR. For the IPRE group, 3 consecutive cycles of 5 min of occlusion and 5 minutes of reperfusion of the coronary arteries were performed before ischemia. For the IPOST group, 3 consecutive cycles of 5 min reperfusion and 5 minutes of occlusion of the coronary arteries were performed before reperfusion. Pathological changes of lung tissue, lung wet-to-dry (W/D) weight ratio, inflammatory factors, oxidative stress indicators, apoptosis of lung cells and endoplasmic reticulum stress (ERS) protein were used to evaluate lung injury.

**Results:**

After myocardial IR, lung injury worsened significantly, manifested by alveolar congestion, hemorrhage, structural destruction of alveolar septal thickening, and interstitial neutrophil infiltration. In addition, lung W/D ratio was increased, plasma inflammatory factors, including interleukin (IL)-6, tumor necrosis factor (TNF)-α, and IL-17A, were increased, malondialdehyde (MDA) activity of lung tissue was increased, and superoxide dismutase (SOD) activity was decreased after myocardial IR. It was accompanied by the increased protein expression levels of ERS-related protein glucose regulatory protein 78 (GRP78), CCAAT/enhancer-binding protein (C/EBP) homologous protein (CHOP), and caspase-12, and the increased apoptotic indices of lung tissues.

**Conclusion:**

IPOST can effectively improve myocardial IR-induced ALI by inhibiting ERS-induced apoptosis of alveolar epithelial cells.

## INTRODUCTION

Under normal physiological and many pathological conditions, there is a high degree of interaction between heart and lung. Acute lung injury (ALI) is a serious complication of heart surgery and acute myocardial infarction^[1,2]^. Acute respiratory distress syndrome is the most serious form of lung injury, which significantly increases mortality, medical expenses and length of hospital stay^[3]^. Myocardial ischemia-reperfusion (IR) injury occurs during heart surgery and revascularization treatment of acute myocardial infarction^[4]^. The heart is one of the most vulnerable organs to ischemic injury. Reactive oxygen free radicals, white blood cell activation and systemic inflammatory response during myocardial IR can cause ischemic microcirculation damage and distal organ damage. Lung is the earliest and most obvious organ with distal organ damage after reperfusion. Myocardial IR will cause ALI to increase morbidity and mortality in patients undergoing heart surgery^[4,5]^. However, there is no specific and effective treatment for ALI after myocardial IR. Therefore, clarifying the mechanism of ALI after myocardial IR is of great significance for searching effective therapies to prevent or treat lung injury.

Endoplasmic reticulum (ER) is an important organelle in eukaryotes, which is also the main site for intracellular protein synthesis, processing, folding, transportation, and intracellular calcium storage. ER can perceive the changes in the intracellular environment over time and maintain its balance^[6]^. When subjected to external stimuli, misfolded and unfolded proteins accumulate in the ER cavity, which activates the unfolded protein response (UPR) to induce endoplasmic reticulum stress (ERS). ERS can lead to pathological imbalance of ER homeostasis and physiological dysfunction^[7]^. Infection, hypoxia, starvation, oxidative stress, calcium disturbance and other stimuli can induce ERS activation, which in turn activates related signaling pathways to induce cell death, inflammation and apoptosis^[8]^. There is much evidence that ERS is involved in lung injury caused by various factors and plays an important pathophysiological role in the occurrence and development of ALI^[9,10]^. Moreover, inhibition of ERS can effectively reduce lipopolysaccharide-induced ALI^[11]^. ALI after myocardial IR is related to inflammation, oxidative stress and autophagy. However, it is not clear whether ERS is involved.

Nonfatal transient ischemia-induced IPRE can produce ischemic tolerance and protect cardiomyocytes from damage after subsequent fatal transient ischemia. However, IPRE occurs before myocardial ischemia, and its clinical application is limited^[12]^. As a new endogenous myocardial protection strategy, IPOST has become a research hotspot due to its organ adaptation to combat IR injury^[13]^. It is a series of transient protective treatments implemented during reperfusion. Its myocardial protection is equivalent to IPRE with clinical feasibility. IPOST can be used as an alternative strategy to IPRE. The biological protective effect of IPOST on the myocardium has been well confirmed in experimental models of a variety of major human diseases and multiple clinical trials^[14,15]^. It can exert a myocardial protective effect by inhibiting inflammation, apoptosis, oxidative stress, and cell pathways^[15,16]^.

In terms of lung diseases, the latest research confirms that IPOST can also protect against lung injury after myocardial IR^[17]^. However, its specific mechanism has not yet been elucidated. Therefore, the purpose of this study was to explore the effect of ischemic postconditioning on ALI after myocardial IR.

## METHODS

### Animals

This study was approved by our hospital's animal experimental medicine ethics committee. All experimental procedures were carried out in accordance with the guidelines for the care and use of laboratory animals. Forty nonpathogenic C57BL/6 male mice, weighing 20-25 g, were purchased from Beijing Weitong Lihua Experimental Animal Technology Co., Ltd. (Beijing, China), No SCXK (Jing) 2016-0010. The mice were maintained in SPF Laboratory of Experimental Animal Center of Xinjiang Medical University at (22±2) °C, relative humidity of (50-60)% and a 12-h light-dark cycle.

### Protocol

**Supplementary Fig. 1:**
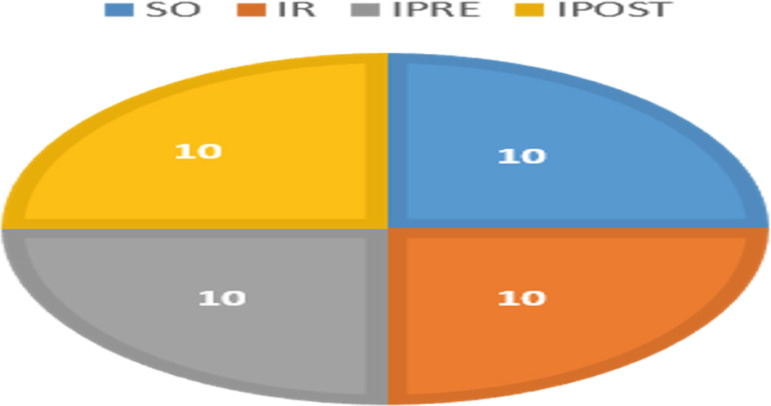
A diagram of the different group stratification.

Forty adult male C57BL/6 mice were randomly divided into a sham operation group (SO group), myocardial ischemia-reperfusion group (IR group), ischemic preconditioning group (IPRE group) and ischemic postconditioning group (IPOST group) (10 mice in each group) (Supplementary Figure 1). Anterior descending coronary artery was blocked for 60 minutes and then reperfused for 15 minutes to induce myocardial IR. For the IPRE group, 3 consecutive cycles of 5 minutes of occlusion and 5 minutes of reperfusion of the coronary arteries were performed before ischemia. For the IPOST group, 3 consecutive cycles of 5 minutes of reperfusion and 5 minutes of occlusion of the coronary arteries were performed before reperfusion.

During anesthesia, a heating pad was used to keep the body temperature between 36.5°C and 37°C. Ketamine (8 mg/100 g), methylthiazide (2 mg/100 g) and atropine (0.12 mg/100 g) (*i.e.*, KXA mixture) were injected intraperitoneally to anesthetize mice, and the injection dose was 0.1-0.2 ml/10g. The electrocardiographic electrodes were connected subcutaneously to the limbs, and 20# venous indwelling needle was used for tracheal intubation under direct oral vision, with a depth of 1.5-2 cm. The tracheal intubation was connected to the small animal ventilator, with a tidal volume of 0.8-1.0 ml and a respiratory rate of 90-110 times/min.

After fixation, the pleura was cut into along the left 3^rd^ and 4^th^ intercostal spaces to enter the thoracic cavity. The intersection point between the pulmonary conus and the right edge of the left atrial appendage and the line between the apices of the heart were used as markers of the anterior descending branch of the coronary artery in mice. The left anterior descending branch was searched under a microscope. The needle is inserted 1-2 mm below the root of the left atrial appendage with a 6/0 suture and the root of the vessel is ligated with the needle from the left edge of the pulmonary artery cone. The sign of successful ligation includes that the movement of the myocardial tissue around the anterior wall of the left ventricle and the apex is weakened, the ST segment of the electrocardiogram (ECG) is elevated by more than 0.2 mv, the T wave is high and the QRS wave is increased and widened. After 60 min, the ligation line was released, and the reperfusion time was 15 min. ECG showed ST-segment depression and redness of the apex.

### Lung Wet-to-Dry Weight Ratio

At the end of the experiment, the left lung of 5 mice in each group was weighed. The wet weight was taken. After dried in an oven at 60°C for 48 h for dehydration, it was weighed again. The dry weight was taken. The lung wet-to-dry (W/D) weight ratio was calculated. The body weight ratio was calculated twice as an index of pulmonary edema.

### Pathological Examination of Lung Tissues

At the end of the experiment, the right upper lobe of the lung of 5 mice from each group was fixed in 10% paraformaldehyde and embedded in paraffin. After cutting into 5 µM slides, hematoxylin-eosin (HE) staining was used to detect the degree of tissue damage. Each animal was randomly divided into 5 sections (3 areas per section). Histopathological evaluation was performed by the blind method. Experienced laboratory pathologists comprehensively evaluated according to alveolar congestion, hemorrhage, infiltration or aggregation of neutrophils in the alveolar or vascular wall, and alveolar wall/hyaline membrane thickness. The four-point system score was used to assess^[1]^: 0, no change; 1, light damage; 2, moderate damage; 3, severe damage. The total score was obtained by adding the characteristic values of each mouse in the group.

### Determination of Interleukin-6, Tumor Necrosis Factor-α, and Interleukin-17A in Plasma

After reperfusion, blood of each group of mice was taken to measure levels of inflammatory factors. Microsample multi-index flow cytometry protein quantification technology and cytometric bead array (CBA) mouse kit (BD company, 560485, USA) were used to determine plasma levels of interleukin-6, tumor necrosis factor-α (TNF-α), and interleukin (IL)-17A. All steps involved were followed the manufacturer's instructions.

### Determination of Malondialdehyde and Superoxide Dismutase Activities in Lung Tissues

Right lung specimens from 10 mice in each group were taken. The microplate reader was used to measure MDA and SOD values in lung tissues. The lung tissue samples were crushed and ground in a mortar and weighed. Phosphate buffer solution (PBS) (4°C) was added according to a mass-to-volume ratio of 1:9 and the mixture was homogenized on ice. The homogenate was centrifuged to collect the supernatant and used as a sample for subsequent experiments. According to the manufacturer's instructions (Nanjing Jiancheng, A003-1), the prepared samples were mixed with vortex mixer and soaked in water at 95°C for 40 min. After cooled with running water, it was centrifuged for 10 min (2000 rpm/min). The absorbance of 300 µL of supernatant was measured at 532 nm for malondialdehyde (MDA) values. According to the manufacturer's instructions (Nanjing Jiancheng, A003-1), the prepared samples were evenly mixed, incubated at 37°C for 20 min, and the absorbance values of each sample were measured at 450 nm for SOD values.

### *In Situ* Detection of Apoptotic Cells in Lung Tissues

Formalin was used to fix, and paraffin was used to embed the lung tissue sections. Then they were used to detect the apoptotic cells in lung tissues that were detected by *in situ* apoptosis detection kit (MK1020, Boster Bioengineering Co., Ltd., Wuhan, China). According to the manufacturer's instructions, terminal deoxynucleotidyl transferase (TdT)-mediated nick-end labeling (TUNEL) method was used to detect and quantify apoptosis. The TUNEL positive cells showed brown stained nuclei under light microscope. Ten lung sections of each mouse were randomly counted by the blind method. At least 100 cells were observed in each field under 200× microscope. The apoptosis index (AI) was calculated as the percentage of stained cells, *i.e.* AI = number of apoptotic cells / total number of nucleated cells × 100%. Apoptosis index was used to measure the degree of apoptosis.

### Western Blotting

After the experiment, the right lung of 3 mice in each group was removed. After rapid freezing in liquid nitrogen, it was placed in the refrigerator at -80°C for standby. About 100 mg of lung tissue sample was taken, and 400 µl of RIPA lysate (Beyotime Institute of Biotechnology, Shanghai, China) was added. After grinded, the mixture was homogenized and centrifuged at 4°C for 15 min at 12,000 rpm. The supernatant was collected to determine the protein concentration by bicinchoninic acid (BCA) protein concentration assay kit (Beyotime Institute of Biotechnology, Shanghai, China). An appropriate amount of 5 × sodium dodecyl sulfate-polyacrylamide gel electrophoresis (SDS-PAGE) buffer was added and heated with boiling water at 100°C for 5 min. The equivalent protein samples were separated by 15% SDS-PAGE and transferred to the polyvinylidene fluoride (PVDF) membrane. The membrane was blocked with 5% skimmed milk powder and incubated with anti-GRP78 (1:500, Abcam, ab21685), anti-C/EBP homologous protein (CHOP) (1:200, Abcam, ab11419), anti-caspase-12 (1:500, Abcam, ab62484) or anti-β-actin antibody (1:800, Shanghai Shenggong, d110001), respectively. After washing with TBST, horseradish peroxidase (HRP)-labeled secondary antibody (ZSGB Biotech Co., Ltd. Beijing, China) was added and incubated at 37°C for 1 h. Finally, the membrane was detected and photographed with Chemiscope minichemiluminescence instrument. Semiquantitative analysis was performed.

### Statistical Analysis

All experiments were repeated three times with the same sample. Statistical analysis was performed using SPSS 22.0 software (IBM Corp., Armonk, NY, USA). Significant differences between groups were assessed by one-way analysis of variance (ANOVA). All data were expressed as mean±standard deviation (SD). Differences were considered statistically significant when *P*<0.05.

## RESULTS

### IPOST Improved the Morphological Characteristics of Lung Tissues

**Fig. 1:**
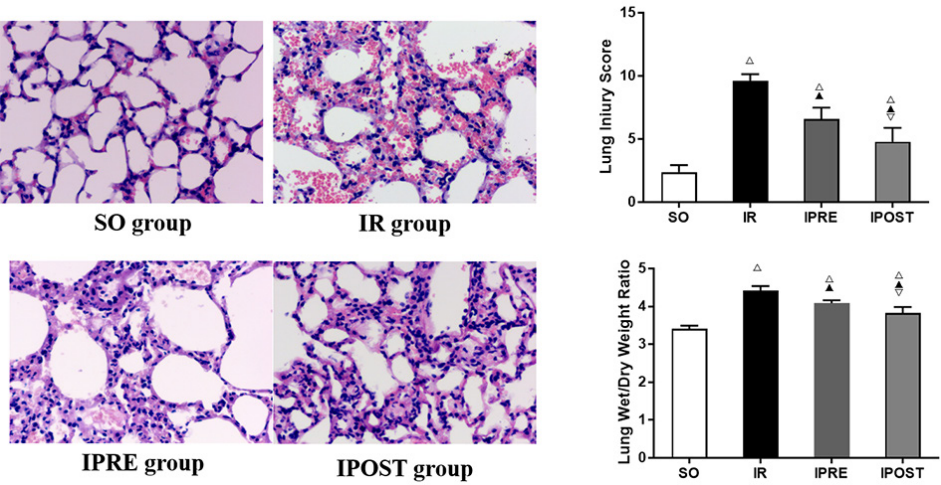
Effect of IPOST on the pathological changes and W/D ratio of lung tissue after myocardial IR. (A) Representative photomicrographs of HE staining of lung sections (200× magnification). (B) Histological score of lung injury. (C) Analysis of lung W/D ratio. Data were expressed as mean±standard deviation (SD). ^△^ compared with SO group, P<0.05; ^▲^ compared with IR group, P<0.05; ^▽^ compared with IPRE group, P<0.05.

Standard HE staining was used to detect the pathological changes in lung tissues under a light microscope. As shown in Figure 1A, there was no obvious pathological change in the SO group. Under the microscope, the alveolar structure of the mice was normal, and there was no narrowing, congestion, hemorrhage, neutrophil infiltration and alveolar septum thickening. In the IR group, there were obvious alveolar congestion, hemorrhage, alveolar septum thickening, structural damage and interstitial neutrophil infiltration. Pathological damages in the IPRE and IPOST groups were significantly reduced. As shown in Figure 1B, the lung injury score of IR group was significantly higher than that of SO group (*P*<0.05), the lung injury scores of IPRE and IPOST groups were significantly lower than that of IR group (*P*<0.05), and the lung injury score of IPOST group was lower than that of IPRE group (*P*<0.05). These results indicated that IPOST had a protective effect on myocardial IR-induced lung injury in mice.

### IPOST Decreased the W/D Ratio of Lung Tissues

At the end of the experiment, the left lung of mice was taken to measure wet weight and dry weight. The effects of IPRE and IPOST on W/D ratio are shown in Figure 1C. Compared with SO group, myocardial IR significantly increased the W/D ratio of lung tissues (4.420±0.119 *vs.* 3.414±0.081, *P*<0.05). Compared with IR group, IPRE and IPOST significantly limited the increase of lung W/D ratio (4.089±0.076 and 3.833±0.154, *P*<0.05). The W/D ratio in the IPOST group was lower than that in the IPRE group (*P*<0.05). These results suggested that IPOST reduced the degree of pulmonary edema after myocardial IR.

### IPOST Decreased Plasma Levels of Inflammatory Factors

**Fig. 2:**
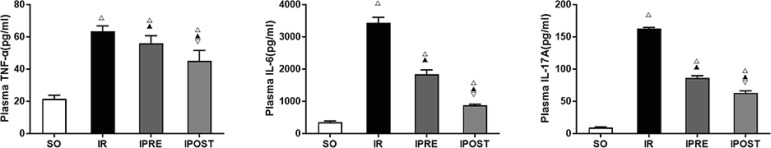
Effect of IPOST on the plasma pro-inflammatory cytokine content after myocardial IR in mice. (A) Tumor necrosis factor alpha (TNF-α) levels; (B) Interleukin 6 (IL-6) levels; (C) Interleukin-17A (IL-17A) levels. Data were expressed as mean±standard deviation (SD). ^△^ compared with SO group, P<0.05; ^▲^ compared with IR group, P<0.05; ^▽^ compared with IPRE group, P<0.05.

At the end of the experiment, the levels of TNF-α, interleukin-6 and IL-17A in the plasma of mice were detected to evaluate the degree of histopathological inflammatory reaction after myocardial IR. As shown in Figure 2, myocardial IR significantly increased the levels of TNF-α, IL-6 and IL-17A in plasma in comparison with SO group (*P*<0.05). Compared with IR group, IPRE and IPOST could significantly limit the increase of TNF-α, IL-6 and IL-17A in plasma (*P*<0.05). Compared with IPRE group, the plasma levels of TNF-α, IL-6 and interleukin-17A in IPOST group were notably lower (*P*<0.05). The results showed that IPOST reduced the inflammatory reaction after myocardial IR.

### IPOST Decreased Lipid Peroxidation and Increased Production of Superoxide Free Radicals in Lung Tissues

**Fig. 3:**
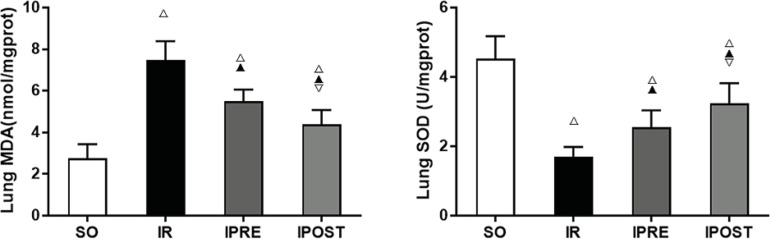
Effects of IPOST on MDA content and SOD activity in lung tissues. (A) MDA level; (B) SOD level. Data were expressed as mean±standard deviation (SD). ^△^ compared with SO group, P<0.05; ^▲^ compared with IR group, P<0.05; ^▽^ compared with IPRE group, P<0.05.

To detect the production of lipid peroxidation and superoxide radical, we measured MDA content of and SOD activity in lung tissues. As shown in Figure 3, compared with SO group, MDA content in lung tissues of IR group increased significantly, while SOD activity decreased significantly (*P*<0.05). Compared with IR group, MDA content decreased and SOD activity notably increased in IPRE and IPOST groups (*P*<0.05). Compared with IPRE group, the MDA content decreased and the SOD activity increased in IPOST group (*P*<0.05). These results suggested that IPOST can reduce lipid peroxidation in lung tissues after myocardial IR but increased the production of superoxide free radicals.

### IPOST Decreased the Apoptosis Rate of Lung Cells

**Fig. 4:**
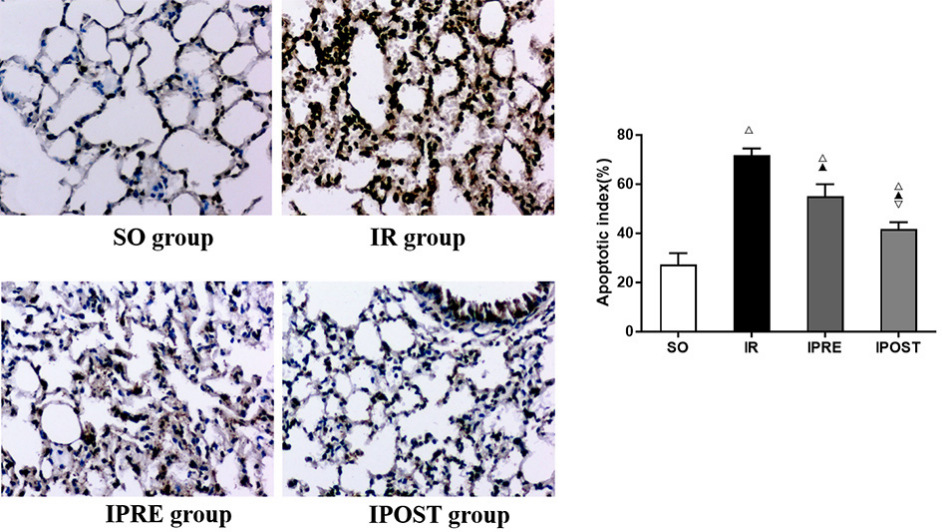
Effect of IPOST on apoptosis of lung tissue after myocardial IR (magnification 200×). (A) Typical micrographs of lung stained with TUNEL. The number of apoptotic cells in lung tissue was significantly increased in IR group and decreased in IPRE and IPOST groups. The number of apoptotic cells in IPOST group was significantly lower than that in IPRE group. (B) The apoptosis index (AI) of lung tissue. Data were expressed as mean±standard deviation (SD). ^△^ compared with SO group, P<0.05; ^▲^ compared with IR group, P<0.05; ^▽^ compared with IPRE group, P<0.05.

To detect the apoptosis rate of lung cells, we performed TUNEL staining. As shown in Figure 4A, the apoptosis in the SO group was lower than in the IR group. In contrast, the number of apoptotic cells in IPRE and IPOST groups was lower than in IR group. As shown in Figure 4B, the apoptotic index of IPRE and IPOST groups were significantly lower than those of IR group (*P*<0.05), while the apoptotic index of IPOST group was lower than that of IPRE group (*P*<0.05). These results indicated that myocardial IR can induce apoptosis of a large number of lung cells, and IPOST can more effectively reduce the apoptosis rate of lung cells.

### IPOST-Inhibited Endoplasmic Reticulum Stress in Lung Tissues

To elucidate the role of IPOST in endoplasmic reticulum stress (ERS), the protein expression levels of (ERS)-related proteins GRP78, CHOP and caspase-12 were analyzed by Western blotting. It showed that compared with SO group, myocardial IR significantly increased the protein expression levels of GRP78, CHOP and caspase-12 in the lung of mice (*P*<0.05). Compared with IR group, IPRE and IPOST limited the increased expressions of GRP78, CHOP and caspase-12 in lung tissue (*P*<0.05). Compared with IPRE group, the protein expression levels of GRP78, CHOP and caspase-12 in lung tissues of IPOST group were significantly lower (*P*<0.05).

## DISCUSSION

Our results suggested that myocardial IR induced ALI by increasing inflammatory response, oxidative stress and ERS-mediated apoptosis, which can be inhibited by IPRE and IPOST. We provided evidence that, compared with IPRE, the postconditioning of three cycles of 5 minutes of reperfusion and 5 minutes of occlusion can provide more effective lung protection, which may be related to the duration of preconditioning and postconditioning and the number of cycles.

ALI is usually caused by a variety of factors, which can increase vascular permeability and inflammatory response. ALI seriously affects the postoperative recovery of patients undergoing cardiac surgery. The mechanism of ALI after myocardial IR is complex and has not been elucidated. Studies have shown that reactive oxygen species, cytokines and prostaglandins can be released out of control after myocardial IR injury. The migration and accumulation of inflammatory factors and apoptotic factors can produce a large number of oxygen free radicals and proteases, leading to injury of pulmonary capillary endothelial cells and alveolar epithelial cells to induce ALI^[18]^. Many pro-inflammatory cytokines, including TNF-α, IL-6 and IL-1β, can be produced after myocardial IR^[19]^. They have been reported to mediate the development of ALI^[20]^. Consistent with previous studies, in this study we provided additional evidence that myocardial IR can cause an increase of circulating pro-inflammatory cytokines TNF-α, IL-6, IL-17A, aggravate lung histopathological injury, increase the lung wet-to-dry weight ratio, lead to increased lung inflammation, and cause ALI.

During the process of reperfusion after myocardial ischemia, a large amount of O_2_ influx leads to the increase of electron leakage of mitochondrial respiratory electron transport chain and neutrophil respiratory burst. These two processes lead to a large number of oxygen free radicals. ROS released from ischemic myocardium may lead to injury of many distal organs^[21]^. Oxidative stress and lipid peroxidation play an important role in distal organ injury after IR^[22]^. The lung is in a hyperoxic environment with a large area and rich blood supply, which is prone to oxidative stress-mediated tissue damage. Effective assessment of oxidative stress can help to more accurately assess the severity of lung injury and predict treatment response and prognosis^[23]^. MDA is one of the end products of lipid peroxidation, considered a marker of cell peroxidation. SOD is an endogenous oxygen free radical scavenger, and its activity can reflect the body's antioxidant capacity^[24,25]^. The results showed that myocardial IR could induce oxidative stress in lung tissues, which showed that MDA levels were increased and SOD levels were decreased in lung tissues. This is consistent with the results of Kip et al.^[22]^.

More and more evidence shows that ERS plays a key role in ALI-induced cell dysfunction^[23,26]^. Because lung epithelial cells secrete a large number of surfactant and other proteins, these cells are prone to ERS^[27]^. ALI can induce ERS-related apoptosis, which is different from the classical apoptosis pathways (exogenous/death receptor and endogenous/mitochondrial cell death pathway). ERS-induced cell death signal can occur through a variety of pathways^[28]^. GRP78 is a central regulator of ER function, a major ER chaperone protein, and is related to the activation of ERS transmembrane sensor. It is a marker protein of UPR and ERS response^[29,30]^. CHOP is an apoptotic transcription factor induced by ERS. It is also a common molecular marker for evaluating ERS. It can hardly be detected under normal physiological conditions. CHOP is significantly induced in ERS and participates in regulating the expression of apoptosis-related genes^[8]^. The recycling of misfolded protein between ER and Golgi complex can enhance the CHOP expression^[31]^. CHOP expression is positively correlated with the severity of ERS, which is the key mediator of ERS leading to cell death^[32]^. Previous studies have shown that CHOP can be activated by UPR signaling pathway, inhibit anti-apoptotic protein, promote the activation of apoptotic protein caspase-3, and induce apoptosis^[33]^. In addition, the typical caspase family apoptosis pathway is unique to ERS. Caspase cascade-related proteins have also been reported as the markers of ERS-related apoptosis. Caspase-12, in particular, is associated with ER membrane and plays a proximal regulatory role in ERS-induced caspase activated apoptosis. Activated caspase-12 can further induce caspase-3 activation, start caspase cascade reaction and lead to apoptosis^[34]^.

Bi et al. reported that ERS proteins CHOP, GRP78 and caspase-12 were significantly increased in lipopolysaccharide (LPS)-induced acute lung injury model, and helix B surface peptide could significantly limit the increase of ERS-related proteins and reduce lung tissue-related apoptosis^[35]^. Consistent with other reports, we found that the expression of ERS-related proteins GRP78, CHOP and caspase-12 in lung tissues were increased and the apoptosis indexes in lung tissues were increased in ALI model after myocardial IR.

IPRE and IPOST, as an endogenous protective pathway, have a significant protective effect on myocardial IR injury^[36]^. However, the application time of IPRE is difficult to control, which limits its clinical application. IPOST overcomes the above shortcomings and is easier to operate and accurately control the time, so it has great potential in clinical application^[37-39]^. Studies have shown that pulmonary ischemic postconditioning is a repetitive injury to the inferior pulmonary vessels, which may aggravate endothelial dysfunction^[40]^. Myocardial ischemic postconditioning can be used as an alternative strategy to protect the lung from IR injury during cardiac surgery without affecting the inferior pulmonary vessels^[17]^. Our study confirmed that myocardial ischemic postconditioning can effectively reduce lung injury, which indicated that myocardial ischemic postconditioning not only protects the heart from the invasion of IR injury, but also protects the distal organs during myocardial IR. This is consistent with the results of Gao et al.^[17]^. Our results showed that ischemic preconditioning and postconditioning could reduce the levels of TNF-α, IL-6 and IL-17A, limit the increase of MDA and decrease the level of SOD in lung tissues after myocardial IR. It could significantly inhibit the activation of ERS-related molecules such as GRP78, CHOP and caspase-12 in lung tissues, and reduce the apoptosis of lung cells. Liu et al.^[41]^ and other studies showed that IPOST and IPRE had the same effect in reducing lung injury in ALI model after intestinal IR. The results showed that the effect of IPOST was more significant than that of IPRE in ALI model after myocardial IR in mice. This may be related to the number of cycles, operating time and model making.

## CONCLUSION

In conclusion, this study confirmed that IPOST and IPRE can reduce myocardial IR-induced lung injury, and its potential mechanism may be related to the inhibition of ERS-mediated apoptosis. In addition, compared with pretreatment, post-treatment not only has advantages in operation and clinical application, but also has a more significant lung protection effect. Our results better elucidated the protective effect and mechanism of IPOST and may provide a theoretical basis for the prevention of lung injury in cardiac surgery and myocardial infarction. Myocardial ischemic postconditioning, as a protective measure superior to preconditioning and pulmonary ischemic postconditioning, has great potential in clinical cardiovascular surgery. However, the specific molecular mechanism still needs further study.

## Figures and Tables

**Fig. 5:**
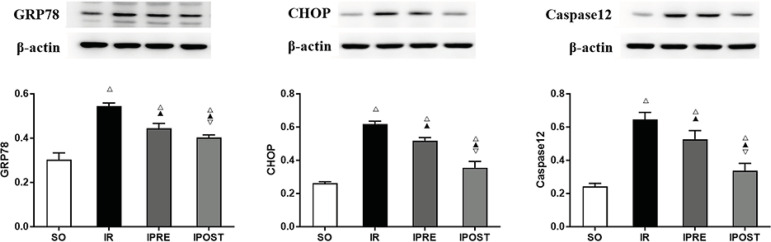
Effect of IPOST on ERS-related proteins in lung tissue after myocardial IR. Western blot was used to analyze the representative results of GRP78, CHOP and caspase-12. (A) Expression of GRP78; (B) CHOP; (C) Caspase-12. ^△^ compared with SO group, P<0.05; ^▲^ compared with IR group, P<0.05; ^▽^ compared with IPRE group, P<0.05.
